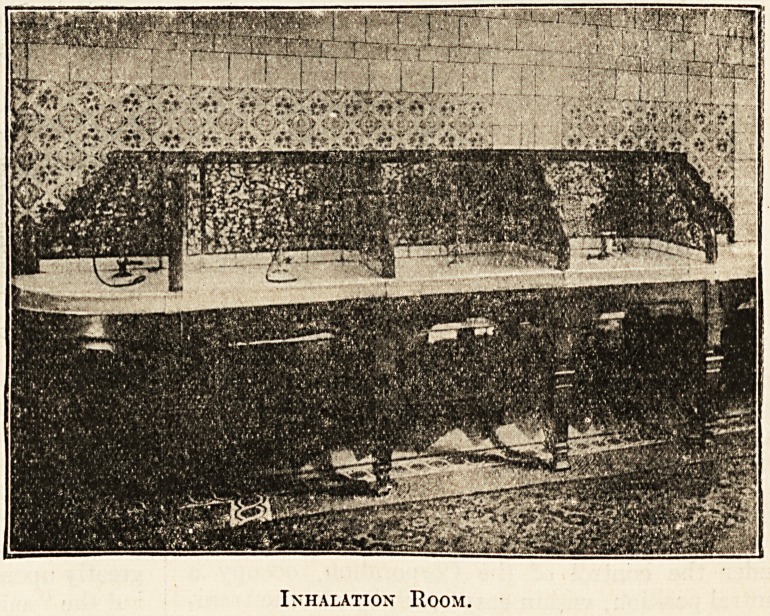# Bath

**Published:** 1911-01-28

**Authors:** 


### HOME AND CONTINENTAL SPAS.

### I.—BATH.

BATH is probably the best known, at least in name, of the spas in this country. "The Queen City of the West" is one of the most ancient, and certainly one of the handsomest, cities in England, and singularly fortunate in its setting, resting as it does in the valley of the Avon, amid the picturesque beauty and vivid green of surrounding hills. Its eighteenth-century streets, circuses, and crescents are unusually imposing, and offer many ideas to those who are interested in the beautifying of our modern cities and towns.

### BATH SPA.

The spa is situate in N.E. Somerset, and is within two hours' reach of London by the Great Western Railway, whose service of trains is both comfortable and adequate, several being fitted with restaurant cars, a convenience in travelling which, for invalids, can scarcely be over-estimated. Many places of historic interest and some of the best scenery in Somerset are to be found in the immediate neighbourhood of the city.

THE GREAT ROMAN BATH.

### POPULATION, AREA, ALTITUDE, CLIMATE.

Bath has a population of 50,000 inhabitants, and at present covers an area of 3,382 acres (of which forty-four are water), but its boundaries are shortly to be increased. The mean altitude is 285 feet, being about 220 above London. The heights of Combe Down on the south and Lansdown Hill on the north are 550 and 750 feet respectively above the sea-level. Within a comparatively circumscribed area Bath possesses a great variety of climate. Central Bath has an equable climate, genial in the coldest weather, and eminently suitable for invalids and elderly people. Observations taken at the Central Climatic Station, Henrietta Park, for ten years give a mean temperature of 49° F. The mean winter temperature is 41°, spring 52°, summer 59°, autumn 46°. February is the coldest month, the average mean temperature being 39°, and July the hottest, with an average of 61.5°.

INSTITUTION GARDENS AND RIVER AVON.

### THE SEASONS AT BATH.

The mean annual rainfall, from observations extending over forty years, is 30.48 inches. The greatest amount in any year was 42.57 inches in 1903, the least amount 21.78 in 1870. The month with the highest monthly average is October, 3.05 inches, whilst the lowest is in May, 2.03 inches. Compared with the N.E. of England, Bath has fifty-one hours more "possible sunshine" in winter and fifty-eight hours less "possible sunshine" in summer. The records for ten years show an average of 243 hours "bright sunshine" during the winter months, 557 hours during the spring, 576 during the summer, and 199 during the autumn months, a yearly average of 1,576 hours of "bright sunshine."

There is a spring and autumn season, but efforts are being made to develop a summer season. June and July are usually perfect months in and around Bath, and at this time the city is thronged with American and Colonial visitors, who do much to add to its gaiety and life. To provide pleasant surroundings for summer visitors, the Corporation has erected in the Institution Gardens a colonnade and fountain, where the mineral waters are served in the open air, which has always been recognised as one of the most attractive features of Continental spa life. The grounds are beautifully situate on the southern bank of the Avon, and here will be found an excellent band, shady trees, gay flower-beds, and comfortable chairs —in fact, a more delightful spot on a summer's day it would be difficult to find. From the gardens there is an exceptionally good view of Pulteney Bridge. This bridge, designed in 1771 by Robert Adam, spans the Avon with three stone arches, over which are built houses and shops.

Grand Pump Room.

### History.

As a spa Bath has been famous for about two centuries, but no doubt exists that the healing virtues of its springs were recognised in the first century by the Romans, who constructed a magnificent series of baths, the remains of which are to be seen around the springs to-day, and are unquestionably the finest and most preserved in Western Europe.

It was in the eighteenth century, however, under the social administration of Beau Nash, that the fame of Bath as a modern spa became world-wide. The first pump-room and many other of the stately buildings which remain to this day were built under the auspices of this famous master of ceremonies, whose exceedingly witty rules may be read there still. In the present pump-room Queen Charlotte held levées during her residence in Bath in 1817, and it was at this time that Bath reached the height of its favour as a health-resort of wealth and fashion.

### The Springs: Their Nature and Constituents.

The hot springs, of which there are three, yield daily over half a million gallons at a temperature of 117° to 120° F. There is consequently, no need for the employment of artificial heat at the risk of the loss of the therapeutic power of the waters.

Bath is the only place in Great Britain where hot springs are found, and the problem of their origin has so far baffled scientific research, although it is believed that radio-activity is in some way responsible for the high temperature they possess. Argon and helion have been separated from the gases of the springs, and the Hon. J. R. Strutt discovered radium both in the waters and their deposits. The radio-activity of Bath water is probably as great as that of any British Spa. Recently Sir James Dewar has demonstrated the existence of the extremely rare gases of krypton and xenon. The waters have been submitted to numerous exhaustive analyses, but no appreciable change in their constituents has ever been recorded.

Reclining Bath.

The following analysis was recently made by the *Lancet*:—

| | Grains per gallon. |
|---|---|
| Calcium sulphate ... ... ... | 102.880 |
| Strontium sulphate ... ... ... | 2.030 |
| Sodium sulphate ... ... ... | 23.500 |
| Potassium sulphate ... ... ... | 0.207 |
| Calcium carbonate ... ... ... | 8.750 |
| Magnesium chloride ... ... ... | 15.800 |
| Sodium chloride ... ... ... | 9.080 |
| Lithium chloride ... ... ... | 0.120 |
| Silica ... ... ... ... ... | 1.960 |
| Bromine ... ... ... ... ... | Traces. |
| Nitrates ... ... ... ... | Traces. |
| Carbonate of iron ... ... ... | 1.600 |
| Total mineral matters ... ... | 165.927 |

### Diseases Treated.

In one or other of the many forms of their application the mineral waters have been found beneficial in cases of rheumatism (chronic and muscular), gout in all its forms, sciatica and lumbago, rheumatoid arthritis, diseases of the skin, disorders of the digestive organs, muco-membranous colitis, anæmia, disorders of the nervous system (neurasthenia and neuritis), tropical diseases, restoring movement and muscular tone after accidents and gunshot wounds, results of injury, etc.

### The Baths.

The extensive bathing establishments, which are under the control of the Corporation, occupy a central position, within easy reach by electric tramcars from all parts of the city. The stations of the Great Western and Midland Railways are only a short walk, and, in addition to electric trams, there is, for those who may need them, no lack of taxicabs, flys; and last, but not least, the world-renowned Bath chairs, which can be hired from ranks in almost all the principal thoroughfares.

The Grand Pump Room is the popular rendezvous of visitors to Bath, and it is here that the hot mineral water is served for drinking direct from the King's Bath Spring. A small orchestra plays every morning from 11.30 to 12.30, which is the fashionable hour for drinking. At this time this finely proportioned eighteenth-century salon is thronged with water-drinkers taking the "cure." The suites of baths have the most up-to-date apparatus, and comprise practically every form of bath known to modern medical science, rendering it possible to administer the mineral waters in from thirty to forty different ways, including Aix massage douche, Needle Barthollet and natural vapour, Bourban-Lancy, Sitz, Nauheim, electric water, Greville electric hot-air baths, and for the more robust and those for whom free movement of the limbs is recommended, swimming baths are provided.

Aix Douche, with Massage.

There is also an inhalation room, fitted with elaborate appliances for spraying the throat, ears, and eyes with mineral waters, in conjunction with such other solutions that may be prescribed, whilst the comfortable private dressing and luxurious cooling rooms ensure every possible comfort for the invalid. The cost of the treatment depends very greatly upon the number and kind of baths required, but the "cure" can be taken for the modest sum of 7s. 6d. per week. The bathing establishments are open all the year round, and the waters are equally efficacious at any period of the year.

This year, by the installation of the Zander methods of treatment, at the Zander Institute, adjoining the New Royal Baths, an extremely important addition has been made to the bathing establishment. This treatment, as is well known, consists of the best-thought-out methods for strengthening and controlling muscular action by means of mechanical apparatus. The Zander machines were designed by Dr. Zander, of Stockholm, in accordance with the latest developments of medico-mechanical science. They are beautifully graduated so that they can be adjusted according to the strength of the person using them, and an accurate record of the development of the different muscles can be obtained. In no method of treatment are the Zander medical gymnastics more suitable than when combined with mineral-water cures and baths. Bath, it may be added, is the only health-resort in Great Britain possessing a Zander Institute. This mechanical treatment is of the greatest benefit in conjunction with the hot mineral baths in cases of rheumatism, stiffened joints, and many other diseases. This Institute is intended not only for those who seek a cure for some particular disease or deformity, but also for those who lead a sedentary life and wish to keep themselves in perfect health. No patient is received for treatment except by the advice and under the direction of his or her medical adviser. The Zander Institute is open daily, and separate hours are arranged for ladies and gentlemen.

Deep Bath, with Chair.

### Diet.

The very important factor of diet is duly recognised, and diet charts for the guidance of patients undergoing the treatment have been drawn up by the Bath Medical Committee, the special requirements and modifications for individual cases being marked by the doctors.

### Accommodation.

The Spa is well provided with first-class hotels and boarding-houses, which in the luxury of their appointments rival the hotels of the Metropolis. These, however, are only for the well-to-do; but it is possible to secure very comfortable apartments, quite close to the baths, at really moderate charges; and as living expenses are usually low, from 45s. to 50s. will cover the necessary expenditure of a week's residence and treatment.

### Amusements and Relaxations.

Bath has long been valued for the opportunities which are afforded for amusement and social gaiety. The Pump Room Concerts enjoy a status which gives them a

Inhalation Room.

foremost place in the attractions of the city. A monthly dance, especially for visitors, is also held in the Pump Room from October to May. Afternoon concerts are given daily in the Concert Hall of the Roman Promenade; here the afternoon may be spent in historic surroundings with music provided by one of the finest orchestras in the kingdom. The theatre, which is open throughout the year, has recently been entirely remodelled, and has accommodation for an audience of nearly 1,500. It secures bookings with some of the best of the touring companies. There is also the Palace, where variety performances are given nightly throughout the greater part of the year.

Of outdoor amusements there is a fair choice; golf may be played over three links, good boating is to be had on the Avon, croquet, lawn tennis, bowls and archery have their grounds, and hunting may be had with the Beaufort pack. The Corporation has recently started an information bureau, where guide-books, particulars of hotel accommodation and apartments, programme of attractions, lists of excursions, etc., may be obtained without trouble or cost. The able and courteous director, Mr. John Hatton, is always pleased to show visitors over the bathing establishments, and in the case of members of the medical profession, complimentary passes are issued both for the treatment and the other attractions of the Spa.

## Figures and Tables

**Figure f1:**
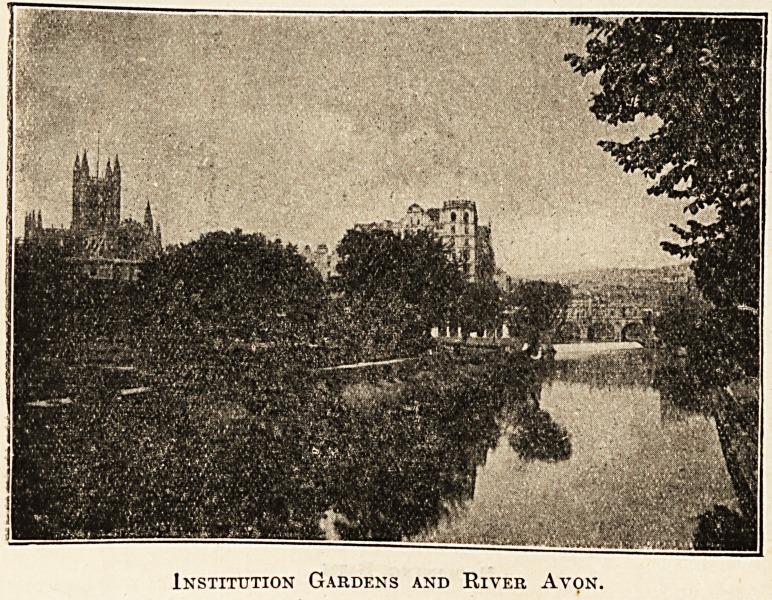


**Figure f2:**
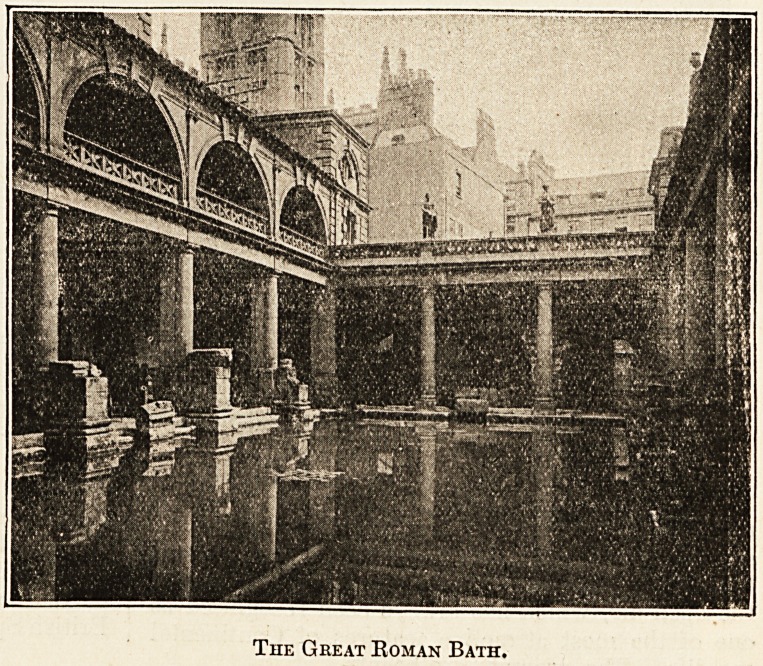


**Figure f3:**
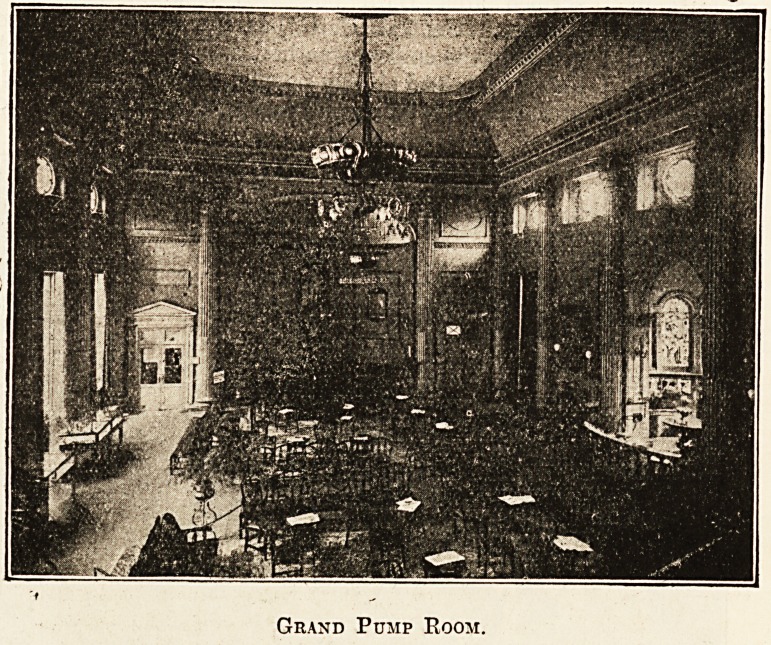


**Figure f4:**
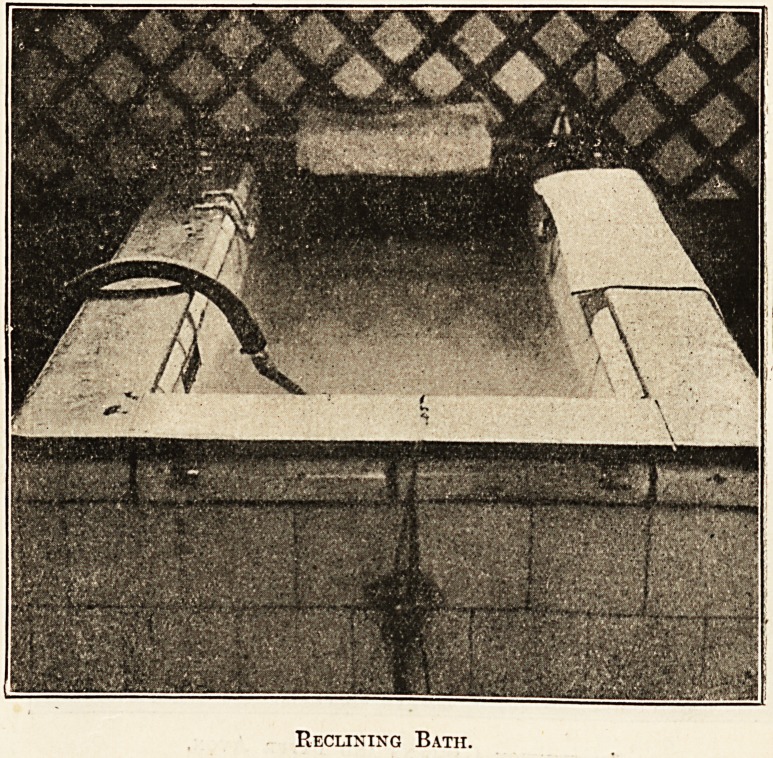


**Figure f5:**
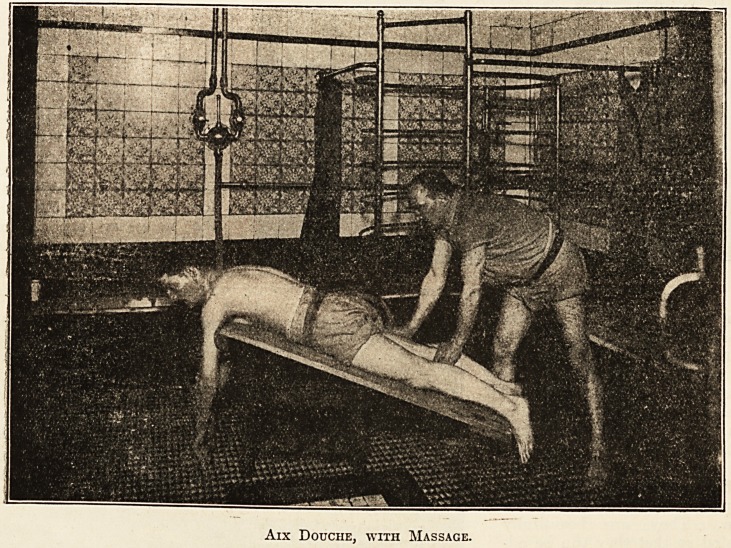


**Figure f6:**
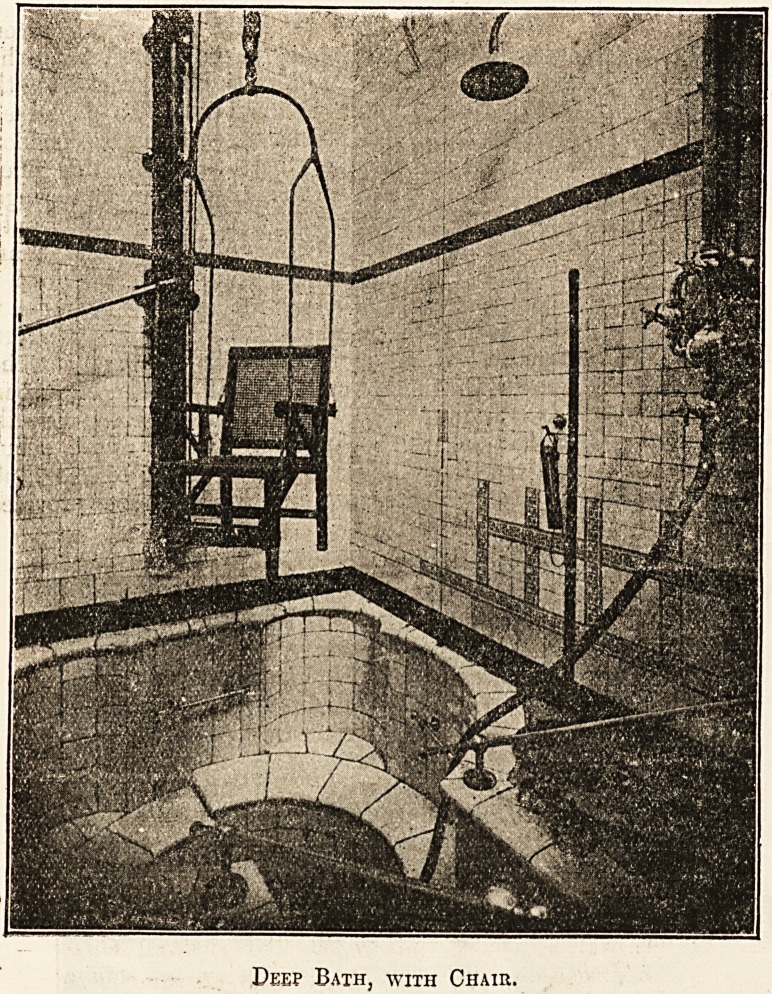


**Figure f7:**